# Struggling with strugglers: using data from selection tools for early identification of medical students at risk of failure

**DOI:** 10.1186/s12909-019-1860-z

**Published:** 2019-11-09

**Authors:** James Li, Rachel Thompson, Boaz Shulruf

**Affiliations:** 1 Faculty of Medicine, University of New South Wales, Sydney, Australia; 2 Office of Medical Education, University of New South Wales, Sydney, Australia

**Keywords:** Medical education, Undergraduate, Australia, Prediction, Medical student

## Abstract

**Background:**

Struggling medical students is an under-researched in medical education. It is known, however, that early identification is important for effective remediation. The aim of the study was to determine the predictive effect of medical school admission tools regarding whether a student will struggle academically.

**Methods:**

Data comprise 700 students from the University of New South Wales undergraduate medical program. The main outcome of interest was whether these students struggled during this 6-year program; they were classified to be struggling they failed any end-of-phase examination but still graduated from the program. Discriminate Function Analysis (DFA) assessed whether their pre-admission academic achievement, Undergraduate Medicine Admission Test (UMAT) and interview scores had predictive effect regarding likelihood to struggle.

**Results:**

A lower pre-admission academic achievement in the form of Australian Tertiary Admission Rank (ATAR) or Grade Point Average (GPA) were found to be the best positive predictors of whether a student was likely to struggle. Lower UMAT and poorer interview scores were found to have a comparatively much smaller predictive effect.

**Conclusion:**

Although medical admission tests are widely used, medical school rarely use these data for educational purposes. The results of this study suggest admission test data can predict who among the admitted students is likely to struggle in the program. Educationally, this information is invaluable. These results indicate that pre-admission academic achievement can be used to predict which students are likely to struggle in an Australian undergraduate medicine program. Further research into predicting other types of struggling students as well as remediation methods are necessary.

## Background

Research into medical student selection often focuses on prediction models for identifying applicants who would excel as students and less so on those who are likely to fail [1]. However, there is a group of students known as ‘strugglers’ who fall into neither of these categories. These struggling students are the ones who encounter academic and/or personal difficulties, which causes them to struggle throughout their study in a medical program [1]. Whilst this is a broad categorisation, often the literature defines such groups differently to one another and uses varying names for the students, such as ‘problem learners’, ‘at-risk students’, ‘disruptive students’, and ‘marginal students’ [2, 3]. The prevalence of strugglers is rarely reported but is believed to lie in the range of 10–15% among undergraduate medical programs [2–4], though rates as high as 36.6–47.3% of students have been reported to have had impeded progress through their medical course [5]. This number could potentially be explained by the usage of different definitions or the fact that struggling students are often unidentified, or poorly reported [2, 6]. These students often report lower motivation, increased frustration and boredom, and lower confidence in their ability to learn [3, 7]. Furthermore, sstruggling has been shown to have a strong association with withdrawal from the course and has also been shown to have significant negative effect upon both the students themselves as well as the universities they attend [1, 3].

Currently, the main challenge posed by these struggling students is the need for effective tools and methods to accurately identify them, so that remediation plans can be put in place [7–10]. These students are not necessarily incapable of completing their course, and in fact, it has been suggested that up to 95% of the struggling medical students do indeed have the essential attributes to successfully complete a medical program [11] . G Paul, G Hinman, S Dottl and J Passon [11] survey, found that students enter medical school with the same level of coping and learning skills, which suggests that struggling students are those who experience more trouble managing their time and the course material. Identifying characteristics specific to students within this group and incorporating them into the medical school selection process could be beneficial as this would allow the implementation of early targeted intervention. Considering the scarcity of knowledge on this topic, research into this area is greatly needed.

The ideal scenario would be to predict those students who may have future difficulties by using data made available through the medical selection process. Previous research suggests that there are a few factors that may be useful for this purpose: lower grades in biology and late offers to the medical program; male gender [12, 13]; lower high school grades [4, 14]; and an inverse relationship between Medical College Admission Test (MCAT) scores and academic difficulty [15]. Some studies reported weak associations between demographic characteristics (e.g. gender, ethnicity etc.) and struggling, but no evidence was found to suggest that factors other than personal attitudes and aptitudes, or previous educational experiences, have any significant impact on the performance of medical students [4].

The group of strugglers is not negligible and too little is known about how best to identify them early in the program. It is therefore suggested that research should be undertaken which aims to: (1) establish a valid set of criteria for identifying struggling students; (2) identify factors that best predict struggling performance; and (3) identify methods for effective intervention. The current study focuses on points (1, 2), whereas point (3) should be addressed in future research.

## Method

### Setting

The University of New South Wales (UNSW) is one of the number of medical schools in New South Wales. Ranking for each admission is based on an average of the student’s overall UMAT score, structured interview, and GPA or ATAR (which is a measure of pre-admission academic achievement). Predictor (independent) variables were the student scores on these three admission tools (Table 1). The outcome (dependent) variable (Table 1) was derived from whether the student had failed any end-of-phase examination but still graduated from the program, but who had still graduated from the UNSW medical program.

**Table 1:** List of variables

Predictor Variables	Description
UMAT	Mean score across all three UMAT sections
ATAR (or equivalent)	Measure of prior academic achievement, either from secondary school (ATAR) or from attendance at a previous university program (GPA)
Interview	Total score of their UNSW admission interview
Outcome variable	
Struggling Status	Whether the student had failed in any of the major course assessment (described below) whilst still completing the UNSW medicine program.

**Fig. 1:**
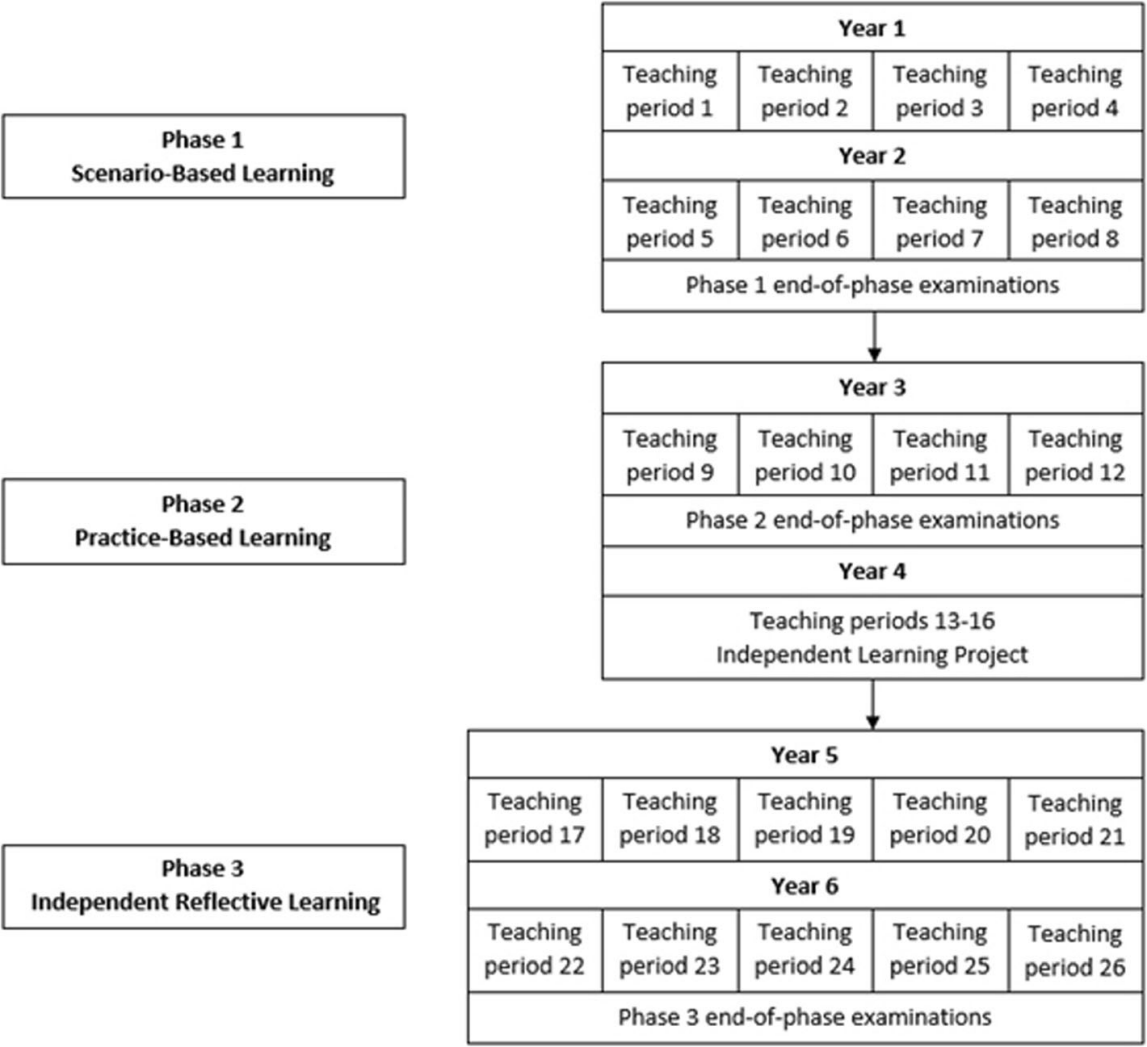
Structure of the UNSW medical program

The six-year UNSW undergraduate medicine program has a modular structure comprising fully integrated courses studies over 26 teaching periods, each of 8 weeks duration (Fig. 1). During each of these courses students must sit course examinations. The program is divided in to three phases based on the approach to learning in each phase. Phase 1 consists of eight 8-week courses in years 1 and 2. Phase 2 consists of clinical courses in Year 3 and the Independent Learning Project in Year 4 (students spend this year undertaking a research project). Phase 3 consists of ten clinical placement courses in years 5 and 6 (Fig. 1). To successfully pass through each phase, students must pass through the end-of-phase examinations: an objective structured clinical examination (OSCE), a portfolio examination, and a written examination [16].

### Study design

The present study examines four consecutive cohorts (commencing years 2006–9) of students that have been through and graduated from the UNSW undergraduate medical program in recent years (admissions and assessment outcomes). We established a data set that combined student’s admission scores and their examination results by cross-referencing their identification numbers. Each student was categorised as struggling or not struggling; struggling students were defined as any student who failed any end-of-phase examination but still graduated from the program. Only the results of the end-of-phase examinations were used as other examinations throughout the program are mostly modular which means that students might undertake them at different times in their course. In comparison, all the students must undertake the end-of-phase examinations at the same point in the medicine program which makes these examinations a more fair and direct comparison between the students. Additionally, students can often proceed with the course and resit a failed course examination at a later point whereas they must successfully pass the end-of-phase exams to proceed. All students that failed during the medical program or dropped out prior to completion were excluded from the analysis. Additionally, no students applying through international pathways were included in the analysis as the admission tools used for those students are different in most cases.

### Study population

There were 700 students in our sample of four cohorts that had both admission scores and examination results. The total study population was 700 UNSW undergraduate medical program students of which 55.3% were female and 44.7% were male. The 700 were comprised of 77.4% local and 22.6% rural students.

### Statistical analysis

We performed a Discriminate function analysis (DFA) to measure how well the current admission tools employed by UNSW were in categorising and differentiating the struggling students from the others. DFA is a statistical procedure that identifies a mathematical function that maximises the accuracy of determining which group a person would belong to, based on the chosen variables [17]. In this study, DFA was used to determine the optimal separation of the students into two groups: a struggling and a not struggling group based on their admission scores. Any student that had to repeat at least one of their end-of-phase assessments or failed an assessment but completed the course was classified as struggling. All other students who graduated from the medicine program without having failed or repeated an end-of-phase assessment were classified as not struggling. The struggling status of the students was compared against their admission scores of ATAR, UMAT and interview scores using the DFA by IBM SPSS v22.0 [18]. The ATAR stands for ‘Australian Tertiary Admission Rank’ which is a number between 0.00 and 99.95 that indicates a student’s position relative to all the students in their age group in NSW in Australia [19]. Applicants/students coming from other states in Australia may have different secondary school results which are then transformed to an ATAR equivalent. UMAT stands for The Undergraduate Medicine and Health Sciences Admission, which is an aptitudes test measuring three domains: Logical Reasoning and Problem Solving; Understanding People; and Non-verbal Reasoning [20].

The DFA generates a function that best discriminates between this subgroup of students and the other students.

### Ethics approval

The research was conducted under the ethics approval granted by the Human Research Ethics Committee ofthe University of New South Wales (reference, HC15421, HREAPG: Health, Medical, Community and Social).

#### Consent

The ethics approval does not require consent from participants (either for using the data for research or for publication), thus such consent was not obtained. This study used administrative data held UNSW Medicine.

## Results

Of the 700 students, 35 were defined to be ‘struggling’ or have struggled during their degree program. Based on the results of the DFA (Table 2), with 73.7% of the cases being correctly classified we proceeded to examine the unstandardized discrimination function coefficients. Based on these results, all three of the admission tools we were investigating were found to negatively predict ‘strugglers’. The use of the three admission tools in predicting which students are likely to struggle produced an odds ratio of 2.05,(95% CI 1.02–4.13, *p* = 0.044). When comparing the standardised function coefficients (Table 3), ATAR had the greatest impact in predicting which students were likely to struggle: the lower the ATAR, the greater the likelihood of the student struggling. The ATAR was found to have a 3–4 times greater impact in predicting who is likely to struggle in comparison to UMAT and interview scores.

**Table 2:** DFA summary table

Classification Results^a^					
			Predicted Group Membership		
		Struggling Status	0	1	Total
Original	Count	Not Struggling	502	163	665
		Struggling	21	14	35
	%	Not Struggling	75.5	24.5	100.0
		Struggling	60.0	40.0	100.0

^a^73.7% of original grouped cases correctly classified

**Table 3:** Efficacy of selection tools in predicting students likely to struggle

Canonical Discriminate Function Coefficients	Standardised	Unstandardised
ATAR (or Equivalent)	0.931	0.570
UMAT	0.219	0.040
Interview	0.297	0.028
(Constant)		−61.110
Functions at Group Centroids		
Struggling Status		
Not Struggling	0.015	
Struggling	−0.290	

The mathematical function produced by the DFA is summarised by the Canonical Discriminate Function Coefficients and the Group centroids (Table 3). Using the corresponding coefficients for each admission factor the optimal cut-score can be produced. Since struggling is a negative value whereas not-struggling is a positive value, any student that falls above the cut-score determined by the DFA would be most likely to be not struggling compared to any student that is below the cut-score. The standardised values allow a comparison of the predictive effect of each admission factor and this shows that ATAR (or equivalent) has the greatest predictive effect of struggling students out of the three admission tools that were analysed.

## Discussion

This study focused on an overlooked, yet important topic in medical student admissions, while determining whether or not selection tools (ATAR, UMAT, and interview scores) had any utility in identifying which students would most likely struggle during an Australian undergraduate medical program. We found that all three admission scores had a slight association, but that the ATAR had the strongest inverse association out of the three admission tools in differentiating struggling students from the other students.

### Comparison to other studies

This study is both novel in the target population we examined as well as in the techniques utilized. Most studies in this area primarily focus on the use of admission tools in identifying students that are most likely to perform well at university, or in identifying those that are likely to fail [21, 22]. This study differs by focusing upon the lesser-researched population of ‘struggling’ students [21, 22]. For the purposes of this study, the focus was on students who struggle academically; defined as any student that successfully graduated from the program but had encountered issues with the course (e.g. failing one or more exams) (see: Method section). The second novel aspect of this project was in the use of DFA for exploring the effectiveness of current medical school selection tools [23].

Other current research regarding admission tools and medical school performance are generally focused on students that excel and students that fail as opposed to looking at the struggling students in between. The consensus of these studies as well as the few that have looked at struggling students is that pre-admission ATAR/GPA is the most effective tool in predicting student performance in the medical program [5, 24–27]. This is often assumed to be the case as ATAR/GPA may be a reflection of either an initial shortcoming in biological concepts and processes, or a shortcoming in their academic ability such as their study skills [28].

### Future policy and research

Our findings raise two main points with respect to the use of these admission tools in admitting students. Firstly, our findings agree with the consensus that previous academic achievement (comprised of either ATAR or GPA) seems to be the most effective admission tool in helping to determine which students should be admitted, but this does question the use of the interview and UMAT with equal weighting to a student’s ATAR or GPA [5, 29]. This is because the other two admission tools have a substantially lower predictive effect than that of the ATAR, though perhaps those two tools are useful in identifying other characteristics that the university finds desirable in its students [16].

The other issue that our findings raise is that the ability of these admission tools to classify these students is neither ineffective nor infallible, but somewhere in between. One question we must consider is whether we can truly say that we can linearly rank/model these students, with the students that do best on the upper end of the scale and the students that fail on the lower end of the scale. Whilst the consensus is that previous academic achievement in the form of ATAR/GPA is the most consistent predictor of medical program success [29], the results of this study suggest that it is not perfect. In that regard, there is the possibility for improvement in terms of admission tools which may allow better ability to identify students that will struggle as well as in creating a more diverse and desirable range of students.

In terms of how these findings could be applied to the medical education and medical programs at various universities, the most straightforward and potentially beneficial way would be to assist struggling students early to prevent future struggling throughout their time in the medical program [9]. Currently, universities can only provide support once a student has already been shown to be struggling (e.g. failing an exam) but based on our results universities could offer targeted support to the specific students who are *likely to struggle*. This is in comparison to the current situation where, if universities wanted to offer early support, it would realistically have to be broad support to the entire student population. By using our findings in this way universities could assist students prior to them struggling and prevent these students from suffering the negative consequences of struggling.

Even though our results show that ATAR/GPA can be used reasonably reliably to identify struggling students, more research needs to be done intotheeffective long-term remediation to assist these students. Additionally, research delving in to the benefits of the use of the UMAT and interviews as admission tools may prove useful as they may in fact be more predictive of other desirable qualities and thus may lead to greater diversity in the medical student and doctor population [25].

### Limitations

The limitations of our study were primarily based on the data we had access to. We were only able to investigate struggling students that had failed in one of the end of phase assessments (as mentioned previously) based on the assessment results we had access to. Because a struggling student was defined as one who failed or had to repeat any end-of-phase assessment but still completed the medicine program, other types of struggling students may have been missed in the scope of this study. Other studies have identified different types of struggling students such as those that may have to postpone or failed assessment because of illness [28] . Additionally, since this study was focused upon the three main admission tools of UMAT, ATAR/GPA and interview scores, many international students who were admitted without the use of all three of these tools were unable to be included within our study. Being unable to assess all possible different categories of struggling students was unavoidable for our project as we were using previously acquired data. For further research, more detailed data and analysis regarding different types of struggling students may provide greater insight in to the predictability of the current admission tools overall.

## Conclusions

The results of this study show that ATAR/GPA is effective in predicting which students are likely to be struggling within the UNSW medical program. Results of this study could be used by other medical schools to assist in early identification of students that are likely to struggle. This would in turn allow the faculty to monitor, track, support, and remediate the students during their time in the medical program. Doing so early is likely to result in better outcomes for these students as well as better prepared and well-equipped physicians entering the medical workforce.

## Data Availability

Data may be available by request submitted to the corresponding author, subject to the approval the Human Research Ethics Committee ofthe University of New South Wales.

## Abbreviations

(GPA): UNSW: University of New South Wales
ATAR: AustralianTertiary Admission Rank
GPA: Grade Point Average
UMAT: The Undergraduate Medicine and Health Sciences Admission Test
